# A Multidimensional Analysis of Religious Extremism

**DOI:** 10.3389/fpsyg.2019.02560

**Published:** 2019-11-18

**Authors:** Susilo Wibisono, Winnifred R. Louis, Jolanda Jetten

**Affiliations:** ^1^School of Psychology, The University of Queensland, Brisbane, QLD, Australia; ^2^Department of Psychology, Universitas Islam Indonesia, Yogyakarta, Indonesia

**Keywords:** radical religion, religion, extremism, religious identity, social identity

## Abstract

Even though religious extremism is currently a hotly debated topic, it is often reduced to a unidimensional construct that is linked to religious violence. We argue that the contemporary use of the term “extreme” fails to capture the different interpretations, beliefs, and attitudes defining extreme religious identity. To address this issue, we unpack the meaning of the term “extreme” in religious contexts and answer the call by scholars to provide a more comprehensive framework that incorporates the many different dimensions that constitute religion. We develop a model of religious extremism in theological, ritual, social, and political dimensions of religion based on the variety of Islamic groups in Indonesia. Going beyond an analysis that equates Muslim extremism with violence, we argue that Muslims (or indeed any religious group) may be extreme in some dimensions but moderate in others, e.g., extreme in ritual and moderate in political. Interpreting extremism relative to these four dimensions provides new insights when examining the global issue of religious extremism and helps to better predict how religious extremism is expressed. More generally, our framework helps to develop an understanding of radicalism that goes beyond a focus on violence.

## Introduction

Recently, we witnessed a surge in psychological research examining the role of religion in human life (e.g., Ysseldyk et al., 2010; Coyle and Lyons, 2011; Brambilla et al., 2016). This rise in interest can probably be explained by recent concerns in the Western world about the social and political implications of the surge of “bad religion” (Basedau et al., 2016). As a result, there has been an intense debate concerning the social risk vs. value of religion and its role within the state (Coyle and Lyons, 2011). Yet, we would argue that the notions of “good” vs. “bad” religions, or even unidimensional and dichotomous categorizations of religiosity as moderate vs. extreme, do not do justice to the issues and lead to simplistic understandings whereby religious extremism is often only seen as a root cause of violence and terrorism.

Such notions and categorizations matter: religious group members are extremely diverse, whereby religion (or even religious extremism) is expressed in very different ways. Problematic too is that such simplistic representations are consequential in that they determine the perception of extremist groups. For example, following 9/11, almost 70% of the U.S. security policies targeted Arabs and Muslims as they were seen to be associated with the adherents of extremist movement (Cainkar, 2009). It appears then that the term religious extremism engenders negative stereotypes toward particular groups of religious people among the public and policy-makers. This occurs despite frequent warnings that popular (or journalistic) uses of the term “extremism,” associating it with terrorism, might lead to misunderstandings of particular groups (Schipper, 2003).

To counter such simplistic understandings of religious extremism, we present a multidimensional model of religious extremism that aims to advance our knowledge of religion as a complex and diverse social identity (Ysseldyk et al., 2010). In particular, we challenge the idea that religious extremism manifests only in one particular way and suggest that one dimension of religious extremism (e.g., a radical agenda in politics) may not necessarily be accompanied by extremism in another dimension (e.g., intolerance for diversity in rituals). To understand people’s willingness to support violent political action, we therefore need to explore religious extremism on multiple dimensions and be open to the idea that not every form of religious extremism is associated with a willingness to achieve goals in violent ways.

In this paper, we briefly review the different interpretations and understandings of extremism within religion and propose an alternative model that allows for a more accurate and complete understanding of various dimensions of religion. We argue that our analysis will help to explain why, despite the perceived similarities that lead outsiders to cluster them together, extreme movements are often in conflict with one another over what it means to be a good religious person. To illustrate the multidimensional nature of religious extremism, we focus on one particular context: Indonesian Muslims. We propose that in other faith contexts, the dimensions proposed may need to be expanded or adjusted to be more accurate and useful.

## Moderate vs. Extreme Religiosity

In psychological research, a variety of terms have been used to describe an engagement with religion, such as religiosity (e.g., Gibbs and Crader, 1970; Diener and Clifton, 2002; Paloutzian, 2017), religious fundamentalism (Altemeyer and Hunsberger, 1992; Williamson, 2010; Liht et al., 2011), radicalism, or extremism (e.g., Simon et al., 2013; Webber et al., 2017; Kruglanski et al., 2018). These terms are sometimes used interchangeably, and sometimes contested (e.g., religious fundamentalism may be used by some scholars to refer to a rigid interpretation of scriptures, in contrast to religious extremism which is often associated with a particular political agenda). Moreover, while religiosity has been linked to positive outcomes such as higher well-being (e.g., Carlucci et al., 2015) and life satisfaction (e.g., Bergan and McConatha, 2001), religious fundamentalism and extremism have been linked to more negative outcomes such as prejudice (Altemeyer and Hunsberger, 1992), hostility (Koopmans, 2015), or even armed conflict (Cornell, 2005).

One prominent definition of extremism as a motivation for terrorism is that extremism comprises ideological beliefs about an obligation to bring back the political system to a form suggested by religious norms through violence (Arena and Arrigo, 2005). Therefore, the label of extremist is attributed to groups fighting for their political agendas against mainstream systems accepted by the majority of people (e.g., ISIS against the government of Syria, or MILF or Moro Islamic Liberation Front against the government of the Philippines). Such a definition of extremism associated with political violence is related to broad collective responses against perceived oppression or injustice, and it may be fueled by extreme religious dogma or not.

The understanding of religious extremism as political has been elaborated by many scholars. For example, religious extremists have been characterized by Sageman (2008) as seeking martyrdom, and fueled by anger regarding perceived injustice. Similarly, Wiktorowicz (2005) proposed a four-stage model of extremism culminating in violence: first, a cognitive openness to new people or new ideas followed by the experiences of personal or group grievance (e.g., discrimination and oppression). Second, the individual takes up activism, and the openness can lead to an acceptance of the group’s extreme norms (e.g., for violence). Belief in the group’s claims and willingness to act based on the group’s norms can overcome the actor’s rational choice perspectives. Thus, when the group’s norm allows the use of non-normative tactics such as violence to gain their objectives, the individuals will intentionally commit violence on behalf of the group.

A similar model of religious extremism as the culmination of a trajectory of religious identity into group-based violence is put forward by Silber and Bhatt (2007). The process of being extreme begins with an openness to new thoughts (e.g., in religion) that leads into a process of worldview change. Within this process, a tragic experience can lead to the loss of meaning and connection with the initial identity (e.g., as a religious moderate). The adoption of extreme beliefs and norms fuelled by tragedy is enhanced by the indoctrination process operated by an extreme organization. Again, religious extremism is seen to reach its ultimate end in the expression of violence by the actor.

The above conceptual approaches to extremism associate extremism with violence committed as a group member. Other more individual-level analyses of extremism operationalize it as endorsement of particular beliefs, such as the duty to engage in violent holy war against the enemy (Webber et al., 2017) or sympathy toward extremist groups and support for their political action (Simon et al., 2013). Some analyses have spanned both individual and group levels: for example, Schmid (2014) proposes that either for individuals (i.e., personal beliefs) or groups (i.e., as embedded in salient group norms), the five warning signs of religious extremism include belief in absolute truth, endorsement of blind obedience, a quest to establish utopia, belief that the end justifies the means, and a declaration of holy war. Similarly, Hogg and Adelman (2013) have defined extremism through the aspects covering group level (i.e., a radical agenda, support for violent action, and authoritarian leadership) and individual level (i.e., extreme pro-group action).

While we applaud the development of more nuanced ways to understand religious extremism, and the diversity of definitions above, we propose that such distinctions do not go far enough in unpacking the multiple ways in which extremism can be expressed. To allow for the development of this diversity, we adopt a broader definition of extremism and define extremism as the extent to which there are clear norms about appropriate behavior and very little latitude in accepting different pattern of norms or particular behaviors. Thus, the focus is not so much on the behavior itself, but on the extent to which particular behaviors are normatively prescribed within a religious group with little room for deviating from that. Therefore, what is perceived as extreme in one historical or cultural context may be moderate or mainstream in another. This usage is in contrast to the definitions proposed by scholars who have associated extremism exclusively with violent intergroup conflict.

In line with Sedgwick (2010), we propose that religion, either at individual or group level, can be expressed along a continuum ranging from moderate to extreme, but go further by arguing that there is not one continuum, but multiple dimensions of religion. We discuss the implication of embracing extremism in one dimension but not in others, and argue that the specific constellations of moderate vs. radical features are important when considering how religion is expressed. To illustrate the multiple dimensions of religious extremism, we focus on the context of Indonesian Muslim groups. We propose that our analysis should also help to understand religious extremism in other faith groups but that the dimensions on which moderate vs. extreme religiosity may be expressed may vary.

## The Multidimensionality of Religion as a Framework to Understanding Extremism

We are certainly not the first to propose that there are multiple dimensions to religion and that these dimensions uniquely connect to important behaviors, such as life satisfaction, stress, youth deviancy. Glock and Stark (1965) suggested that within all religions, there are five distinct components: ideological (beliefs), intellectual (knowledge), ritual (overt religious behavior), experiential (feelings or emotions), and consequential (the effect of religiosity in the world). More recently, Saroglou (2011) proposed four basic dimensions of religion and individual religiosity that are partially distinct: *believing* (i.e., representing the cognitive function of religion), *bonding* (i.e., experiences that bond individuals with perceived transcendent reality, others, and the inner-self), *behaving* (i.e., specific norms and moral arguments defining right and wrong), and *belonging* (i.e., identification with particular tradition, denomination, or a specific religious group). According to these models, and others, the behavioral expression of religion is complex and multi-faceted.

We draw on these frameworks to examine religious extremism. Combining insights from these prior models, we propose a multidimensional structure to religion that can help to understand the ways in which moderate vs. extreme religion can be expressed. We elaborate our four-dimensional model below, but to summarize: our starting point is the literature on violent religious extremism, where the most common dimension identified (and often the only dimension considered) is the political dimension. In addition, we were inspired by various religious movements in Indonesia that have different emphasis on their narratives and actions. For example, a group named *Wahdah Islamiyah* has a strong campaign to purify Muslims’ theological beliefs and the way religious rituals are conducted, seeking to return to an ideal standard of the past. However, they tend to accept the current political system employed to rule the nation. In contrast, *Hizbut Tahrir*, a banned organization in Indonesia, proposes that Muslims are responsible to recreate an Islamic empire, by rejecting democratic systems and nation-states; however, *Hizbut Tahrir* does not typically engage in theological debates. Therefore, alongside the political dimension, we also consider three other dimensions which emerge in seeking to understand religious extremism in Indonesia. A second theological dimension of extremism that is relevant in the Indonesian context is adapted from Saroglou’s (2011) *believing* dimension: we propose that religions share theological beliefs, and these beliefs might be extreme or moderate. In the Indonesian context, a third, ritual, dimension is inspired by Saroglou’s (2011) *bonding* dimension indicating that religion bonds its members through ritual practices. Some groups have very little latitude in how they understand and practice their religious rituals and justify the other practices as forbidden innovation. Finally, we propose a social dimension that captures the intra- and intergroup relations of the religious group in Indonesia. Intra-group processes include the specific group norms that control the members’ moral compass and relations to each other. Intergroup processes include the categorization of in-groups and out-groups as reflected in Saroglou’s dimension of *belonging*, but also the specific group norms controlling relations to members of other faiths.

Before elaborating these dimensions, it is important to note that the four focal dimensions in the present paper do not imply that other dimensions do not exist when explaining religious extremism. We propose that the present dimensions are important in understanding religious groups’ perspective in the contemporary Indonesian context. Yet, these four dimensions may become more or less important as a result of particular historical and cultural developments or group comparisons, and this may also mean that other dimensions may need to be considered for other religious groups, and when studying other contexts (see also, Zarkasyi, 2008; Ysseldyk et al., 2010). Below we consider the four dimensions in turn, and identify how the dimensions might be used in research.

## Moderate vs. Extreme Religion: A Multidimensional Approach Based on Religious Movements in Indonesia

**Figure 1:**
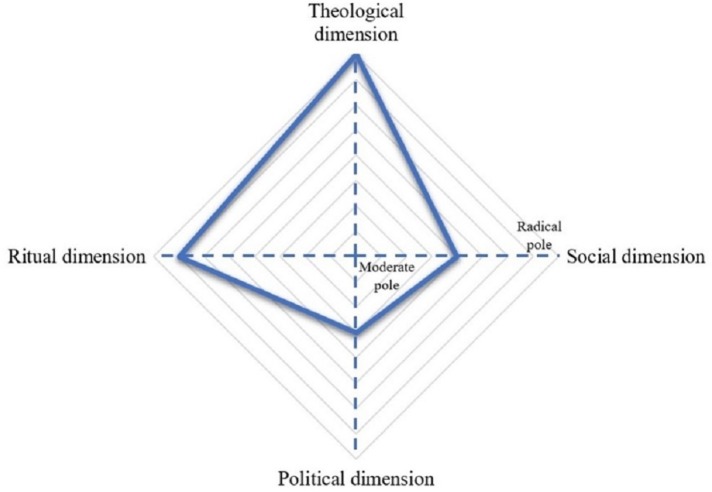
Illustration of the extreme-moderate continuum across a multidimensional representation of religion. The figure illustrates a religious group with high extremism in theological and ritual dimensions but moderate beliefs in the social and political dimensions.

We address religious extremism and the multiple dimensions of religion in Figure 1. Using a classical standpoint that religion is expressed through multiple dimensions (Glock and Stark, 1965; Saroglou, 2011), we propose to examine a multidimensional religious extremism through separate political, theological, ritual, and social dimensions which may or may not co-vary.

We argue that this exercise enables a more nuanced and comprehensive understanding of religious extremism as presented in Table 1. In what follows, we draw attention to the interplay of these different dimensions for different religious groups. We argue that the four dimensions of religious extremism (i.e., political, theological, ritual, and social) provide a useful framework to locate religious groups, which allows for a better understanding of the way in which their religion is expressed and, importantly, the way in which they aim to achieve religious goals by peaceful or less peaceful ways. Following our outline of the four dimensions, we discuss a methodology for measuring context-specific religious extremism and discuss research applications.

**Table 1:** Mapping out the moderate and extreme end points of the four dimensions of religion.

Dimensions	Moderate end	Extreme end
Theological	Main characteristic: gracious theology	Main characteristic: authoritarian theology
	In the context of Muslims:	In the context of Muslims:
	1. Emphasis on God as a loving God and	1. Emphasis on God as an angry God and
	2. A flexible interpretation of “jihad” with connotations to positive change.	2. “Jihad” refers to only a holy war.
Ritual	Main characteristic: tolerance of diversity	Main characteristic: intolerance of diversity
	In the context of Muslims:	In the context of Muslims:
	Viewing the integration of rituals from Islam with local traditions as positive cultural practices.	Actively rejecting local traditions and judging actors involved in them as sinful.
Social	Main characteristic: complexity acceptance	Main characteristic: complexity avoidance
	In the context of Muslims:	In the context of Muslims:
	1. Attributing in-group problems to anti-intellectual biases, geopolitical instability, and corruption and	1. Attributing in-group problems to out-group conspiracies and
	2. Respecting people living with different norms.	2. Judging others by in-group’s specific norms.
Political	Main characteristic: maintenance agenda	Main characteristic: radical agenda
	In the context of Muslims:	In the context of Muslims:
	Integrating Islamic values with modern political concepts.	Advocating for a resurrection of an Islamic empire.

### From Moderate to Extreme: The Political Dimension

The way that religion and politics should relate has been a source of intense debate and struggle throughout history (Armstrong, 2000). In the psychological literature, the political dimension of religion has not received much attention (e.g., Diener and Clifton, 2002; Saroglou, 2010) unless it is in the context of “bad” religion (Basedau et al., 2016). Nevertheless, as noted above, political beliefs and actions have been the primary focus of “religious extremism” as defined by scholars (e.g., Webber et al., 2017). In this approach, extreme religious movements seek political power, to promote the adoption of their religious norms through laws or force.

Contemporary religious extremism in the political dimension for Islam is often associated with support for the Caliphate or Muslim empire, which persisted in various forms from the 700 s to 1924 AD, when the last Ottoman Caliphate was abolished in Turkey. During this time, Islam was associated with both a hierarchical, sometimes expansionist, imperial state and a specific system of religious, legal, and cultural practices called *sharia* law. More extreme groups advocate a radical agenda in political change (e.g., a resurrection of an Islamic empire, a borderless state encompassing all Muslim nations, Ward, 2009; Osman, 2010a). They believe that political norms should be applied to change the current locally adapted political systems across many Muslim countries. Other, more moderate groups, however, strive for an integration of religious values within the current political systems (e.g., democracy, national state, etc.). They tend to believe that religion should not be politically represented through the legalization of its social order.

The political dimension is typically the most salient dimension for scholars when discussing Islamic religious extremism, as it is for other groups. More extreme stances on this political dimension such as support for comprehensive *sharia*, support for the resurrection of the Caliphate, and the rejection of democracy were used by Fealy (2004) to identify extremist groups in Indonesia (e.g., *Darul Islam*, Indonesian Mujahedeen Forum, Jihad troops, and *Hizbut Tahrir*). In addition, the extent to which religious groups approve of and participate in current political systems in Indonesia has been used to identify the more moderate Islamic movements in Indonesia (e.g., *Muhammadiyah*, *Nahdhatul Ulama*, etc.).

It should be noted, however, that religious extremism on the political dimension can manifest in different ways, and that politically extremist groups propose different paths to reconcile the constitution with religious norms. For example, some religious groups propose to promote *sharia* laws through democratic governance, other groups reject the current political system by actively campaigning for the imposition of *sharia* without violence; and finally, still other groups are willing to use violence to destabilize the government (See Ward, 2009; Webber et al., 2017). With or without support for violent means of creating change, the advocacy toward comprehensive *sharia* law as well as the revival of an Islamic empire reflects a radical agenda to transform the current established political system.

In addition to these different views regarding the place of religion in the state, groups of Muslim also differ in their support for democracy (e.g., Halla et al., 2013). Some of them reject democracy, believing that democracy as a political system is incompatible with Islam (Fealy, 2004; Ward, 2009), that Islamic instructions about all matters relating to life are clearly articulated in the Quran and *Hadith* (the words, behaviors, and approvals of the prophet). This view holds that the *musyawara* (political discussions to reach a consensus) should only be used for decisions about particular technical matters, not core principles of social functioning (Nurhayati, 2014). In contrast, some other groups of Muslim do not favor or sanction a particular political system, but rather advocate for principles of tolerance and respect in the governance of all political systems. In this way, the latter groups perceive democracy as one way to manage national affairs that is not in conflict with Islam (Ward, 2009; Nurhayati, 2014).

### From Moderate to Extreme: The Theological Dimension

Theological beliefs define religion for lay people (Saroglou, 2011), and researchers such as Stark and Glock (1968) have highlighted the importance of conceptual representations of God in understanding people’s engagement with their religion (see also, Granqvist et al., 2010). Different conceptualizations of God provide a meaningful snapshot of a believer’s religious worldview. How then do more moderate vs. more extreme forms of religion take shape? We propose that for religious groups that are located at the moderate end of the theological dimension, beliefs of an impersonal cosmic force distanced from worldly affairs (deism) dominate. Moderate views of God as a personal agent (theism; Bader and Palmer, 2011) present a being fostering love and not hostility, whereby the image of God is characterized by gracious images (e.g., The Merciful, The Benevolent, etc.) allowing different religious interpretations and expanding the acceptance toward different patterns of norms. In contrast, groups that are located at the extreme end of the theological continuum typically view God as a personal agent and embrace names for God that contain an authoritarian image (e.g., The Compeller, The Conqueror, etc.) leading into rigid interpretations and coercion to suppress different narratives.

There is evidence that these images of God are consequential. For example, normative beliefs associated with an authoritarian image of God predict more support for capital punishment (Bader and Palmer, 2011). In addition, an authoritarian conception (e.g., God as the One who strikes down in anger) has been found to be associated with a disposition to think, feel, and act more punitively toward people considered to be “evil.” In contrast, people who characterize God in a more nurturing way (e.g., God is love) react in a more prosocial way toward others (Granqvist et al., 2010). Historically, an authoritarian image of God was frequently associated with apocalyptic narratives to attract people to convert into their group and to force people to leave their “immoral” norms (Bossy, 2001).

Building on this approach, we propose that variation on the theological dimension of extremism is associated with different behaviors to achieve group goals and to show loyalty to the religious group. Moderate positions on the theological dimension are indicated by the prominence of gracious images of God and an appreciation of differences in theological beliefs. Conversely, those groups located at the extreme end of the theological continuum, embracing an authoritarian image of God, are more likely to strike at perceived contrary theological beliefs. For example, we propose that those who believe in an authoritarian, persecuting God will be more likely to believe that natural disasters occur more frequently to groups who live in ways that God disapproves of. Furthermore, Muslims who endorse an authoritarian conception of God will tend to define the meaning of “*jihad*” as the duty to engage in a holy war, whereas those located at the more moderate end of the continuum will take a more flexible interpretation of the word “*jihad*” as behavior aimed at creating positive change (Esposito, 2002). A similar distinction can be witnessed in Christianity: interpretations of the religious duty to build the “Kingdom of God” will be interpreted by moderates as a spiritual exercise to transform society toward being more loving, caring, and inclusive, whereas for those Christian groups located at the extreme end of the theological continuum, this duty is seen as a need to build a physical empire established through crusading military ventures (Whitlark, 2011).

### From Moderate to Extreme: The Ritual Dimension

Specific rituals allow for the expression of worship or shared feelings with others (Whitehouse and Lanman, 2014), helping to construe notions of religion as a lived experience. Many religious people believe that their religious rituals are guided directly by God. For instance, most Muslims believe that God directs their main rituals including their prayers five times per day. However, once religion has spread to a wider community, different patterns of rituals may emerge from either local customs or from the integration of religious rituals with local traditions. This accommodation of new practices can often polarize religious adherents into groups who are either open to new influences, or who reject compromises and see them as forbidden innovations. For instance, some Mandinga immigrants in Portugal view a “writing-on-the-hand” ritual as essential for conferring both Muslim and ethnic identities (Johnson, 2006). In the ritual, children are initiated into Quranic study (and adulthood) by having a verse written on their hands, which they then ceremoniously lick off, ingesting the verse. The ritual is contentious to those who feel that this Mandinga “custom” should be abandoned to keep Islam pure (Johnson, 2006).

We propose that intolerance of diversity in ritual practices distinguishes moderates from extreme religious groups on this dimension. Moderate religion on the ritual dimension is indicated by tolerant views about the influence of local traditions on the way rituals are performed. Moderate groups can accept the influence of tradition within ritual as it is not a compulsory ritual and not directly taught by God. Conversely, those groups located at the extreme end of the theological continuum are more likely to strive to keep religious rituals pure. This often goes together with vigilance to protect the integrity of rituals, but also with intergroup tension whereby extreme groups accuse more moderate groups of being sinful in their practice. For example, the *Salafi* movement in Indonesia is the strongest group to strive for purity in rituals opposing more relaxed Muslim religious traditions such as allowing worship in the ancestors’ graveyard and the celebration of the Prophet’s birthday. To some extent, the Salafi movement labels the fellow Muslims who perform those rituals as idolatrous (*Musyrik*) or even infidels (*Kafir*). The labels, of course, are psychologically painful for the labeled groups of Muslims. Within the Christian tradition, the Puritan movement is one historical example of ritual extremism, rejecting other Christian denominations as insufficiently pure and compromised by lax tolerance of cultural practices.

### From Moderate to Extreme: The Social Dimension

The social dimension is concerned with intergroup relations with other groups (religious and otherwise) as well as intragroup processes, reflected in norms regarding how to interact with others. Extremists on the social dimension typically have a hostile view of other faiths. In their view, out-groups use sinister conspirators as pawns to influence their religious group members (Fealy, 2004; Kohut et al., 2006). As a result, blaming others (e.g., foreigners) for in-group disadvantage is a common narrative to raise support from the public for their agenda. In contrast, more moderate members of a religion often attribute the root causes of in-group problems to internal factors such as anti-intellectual biases, geopolitical instability, and corruption (Lackey, 2013). The moderate groups tend to be more open to complexity in analyzing the causes of the in-group’s problems. Moderate groups also place greater emphasis on the need to change to address modern concerns.

Consequently, more moderate groups tend to be more open to collaboration in inter-religious and inter-cultural dialogue whereas more extreme religious groups emphasize rejection and avoidance. Interfaith dialogue is often developed by more moderate religious activists to strengthen inter-religious cooperation as a way to solve common problems (e.g., environmental issues, a cohesive national identity, economic issues, and law enforcement). However, more extreme religious groups often actively reject this collaborative effort, as they perceive inter-religious dialogue as part of a conspiracy to weaken the faith in their religion.

Turning to intragroup relations with other members of the faith community: religion serves as an organizing set of key values that are captured and expressed in group norms. In the context of social relationships, such group norms may vary in the extent to which they tolerate difference and dissent within the religious group. At times, harsh attitudes toward dissenters and deviants may prevail when universal values of tolerance and group-specific values clash and individuals are forced to follow group-specific values. For instance, because Islam forbids liquors, some Muslims would like to force the government to ban the trade in alcohol, without considering that other groups of people have different norms permitting alcohol consumption (Osman, 2010a). In the context of Indonesia, an active group called the Islamic Defenders Front (FPI) is one of many aiming to force the entire nation to follow one version of Islamic social norms (Arifianto, 2017). Hence, the social dimension of religious extremism in our approach is indicated in Indonesia by patterns of externally attributing the causes of in-group disadvantages, and forcing out-groups as well as all in-group members to follow narrow, prescriptive social norms. Naturally, social and political dimensions of religion will often be inter-related, especially where groups seek political power to impose their socially extreme viewpoint. However, in distinguishing the two dimensions, we highlight that some groups will be socially extreme without endorsing extreme political views or seeking political power. Groups who expel internal heretics and who shun contact with infidels without trying to dominate them may fall into this category, in our model.

## The Interaction Among Multiple Dimensions of Religious Extremism in Indonesia

As mentioned, the discourse of religious extremism has mostly been related to the political context (Fealy, 2004; Zarkasyi, 2008). To illustrate the importance of not just exploring the political dimension when understanding religious extremism, we took a closer look at some “extremist” Islamic movements in Indonesia (i.e., that score high in extremism on the political dimension). In an attempt to understand different forms of extremism more comprehensively, we compared these groups on the other three dimensions. Before outlining our findings, it is important to note that the classification of a particular group as politically extreme was based on specific historical events and developments: by acts of political rebellion by *Darul Islam* (Domain of Islam) and *Negara Islam Indonesia* (Indonesian Islamic State) in 1949. This was also the basis for selecting as extreme the current political movement *Hizbut Tahrir* and Islamic defender front (FPI) who have gained support after the reform of 1998 (Fealy, 2004; Muhtadi, 2009; Osman, 2010a).

There are important similarities between *Jamaah Islamiyah* (JI), *Majelis Mujahidin Indonesia* (MMI), and *Hizbut Tahrir* (HT) across all four dimensions of religious extremism. All groups score high in extremism on the political dimensions in that they demand a comprehensive legalization of *sharia*, a fully Islamic state, recreation of Caliphate, and the abolition of democracy in Indonesia. However, these groups differ from other politically “extreme” groups in Indonesia. For example, the Islamic Defenders Front (FPI) supports a comprehensive legalization of sharia, but endorses democracy and rejects the revival of the Islamic state and Caliphate (Fealy, 2004). Another group (*Laskar Jihad* or Jihad Troops) demands comprehensive *sharia* and rejects democracy, but also rejects the revival of the Islamic state and Caliphate. Both these commonalities and differences have consequences for their relationship with other religious groups and the way they aim to achieve their goals. While we acknowledge the importance of unpacking the political dimension into constituent elements in some cases, our argument is that to fully understand these groups, we also need to explore where these groups stand on the other three dimensions of religious extremism (i.e., theological, ritual, and social dimensions).

In terms of extremism in the theological dimension, notions about an angry God who uses natural disasters punitively are particularly important to tease the different extremist groups apart. For instance, some Muslim groups in Indonesia claim that ritual celebration of the local tradition in Palu in Central Sulawesi is a main cause of the earthquake and tsunami that hit the Indonesian coast in 2018, killing more than 2000 people. Likewise, such attributions also dominated when explaining the 2018 earthquake in Lombok Island (Habdan and Baits, 2018). These groups emphasized that the earthquake is a punishment from God to show disapproval of the politically different attitudes that are promoted by the political leader of the Island (Hasan, 2018). Interestingly, such theological beliefs do not lead to a push for change of the political system, but only to an invitation to return to Islamic norms as they understand them. This shows that an extreme theological belief may not be correlated with extremism on the political dimension.

However, extremism in the theological dimension may also be related to a narrow interpretation of *jihad* as a core principle in Islam. Most Muslim groups believe that *jihad* means any zealous effort to bring about a better world (Esposito, 2002). However, some groups restrict its interpretation to waging holy war, such as Jemaah Islamiyah (JI), *Salafi Jihadi* groups, and *Jemaah Ansharut Tauhid* (Haron and Hussin, 2013). Theological beliefs restricting the meaning of jihad to waging holy war have an impact on political extremism, in that these can drive believers into intentions to verbally or physically attack the hated out-groups to engage in jihad.

Finally, some groups that are located at the extreme end of the ritual dimension are actively campaigning to purify religious rituals and to suppress local traditions that are perceived as deviating from Islam. For example, some groups such as the *Salafi* movement and *al Wahdah al Islamiyah* in Indonesia campaign against local traditions and push for the Muslims to relinquish traditions that are perceived as not taught by the prophet (Salman, 2017). Importantly though, these movements do not use physical violence in their efforts, and they accept and participate in the political system in Indonesia. Thus, although these groups tend to be extreme on the ritual dimension, they are more moderate on other dimensions. For example, they have a broader conception of *jihad* (i.e., a struggle for positive change), and they do not prevent their members from participating in the current political system.

We have argued that extremism on the social dimension is represented by the tendency to blame others for the group’s disadvantage and to force compliance to specific in-group’s norms. We propose that the tendency to forcefully demand adherence to a narrow version of the in-group’s norms typically results from feeling threatened by out-groups’ norms. For example, the Muslim Forum of Bogor (FMB) released a public statement calling on the city mayor to ban the celebration of Cap Go Meh by Chinese people in the city. Even though such social extremism often involves intolerance of norm violations, social extremism is not always followed by extremism on other dimensions (e.g., ritual dimension). In particular, social extremism in Indonesia is rarely linked to terror campaigns.

## The Consequences of Similarity in Extremism on Multiple Dimensions for Intergroup Relations

The different ways in which religious groups express their religious identity on the theological, ritual, social, and political dimensions affect not only the ways they aim to achieve their goals but also the ways they relate to other religious groups. Using social identity theory as a lens to conceptualize intergroup relations (Turner and Oakes, 1986), we propose that the nature of intergroup relations between moderate and extreme religious groups is determined by the perceived degree of similarity on the four dimensions. As an illustration, two groups or more can cooperate with each other in their collective action when they perceive shared values and a larger identity, while breaking into conflict when internal differences are salient. For example, in Indonesia, when the former governor of Jakarta (Basuki Tjahaya Purnama aka Ahok) was eventually indicted on charges of insulting a section of the Quran, many Muslim groups were united in their efforts to demand punishment of him. A series of mass protests against the perceived blasphemy were attended by hundreds of thousands of people across the country (Fealy, 2016). From an identity perspective, it can be argued that the shared outrage about the former governor who was perceived to have insulted Islam brought different Muslim groups together, and different groups worked together to address the common grievances and the common threats to the superordinate Muslim identity.

Despite this example of unity, it is also clear that there are many instances when relationships between moderate as well as more extreme religious groups are more tense. We argue that these tensions can also be better understood by taking account of the way in which moderate vs. more extreme expressions of identity take shape on the four identified dimensions. For example, members of The Prosperous Justice Party (PKS) and members of *Hizbut Tahrir* largely take the same stance on the social dimension in that both groups want to generate a new Islamic social order *via* the legalization of *sharia* in Indonesia. However, The Prosperous Justice Party (PKS) frequently criticizes the members of *Hizbut Tahrir* because they disagree with the best “Islamic” method to achieve their shared goal. Their disagreement emerges on the political dimension because PKS supports the democratic system, as indicated by their participation in the general election, while *Hizbut Tahrir* absolutely rejects the democratic system and avoids democratic politics as a way to raise political power.

The possibility of compromise between two politically extreme movements depends on the level of identity (i.e., subgroup or subordinate identity) that is activated. When they confront common enemies (e.g., a group of Muslims or politicians who strongly support Indonesian diversity and oppose the legalization of Islamic law), the salience of their superordinate identity (i.e., as Muslim groups advocating the legalization of Islamic law in Indonesia) may increase, and they may compromise or even integrate. However, open conflict is also likely, even if the groups are similarly extreme on one dimension, when differences on another dimension are salient.

A similar pattern may be observed among groups of Muslims who are identified as extreme in ritual dimensions. The *Salafy* movement and other groups (e.g., *Mathla’ul Anwar*, *Wahdah al Islamiyah*, etc.) may unite to produce narratives for ritual purification, and to accuse Muslims who practice local traditions and their supporters of religious error. That is, when they face moderate Muslims (e.g., *Nahdhatul Ulama*, a group which supports the preservation of local traditions and diversity), they will activate a shared superordinate identity and work together. However, those ritually extreme groups can conflict with each other when political differences are salient. For example, many *Salafy* group members perceive that public protest is an illegitimate action according to Islam, while other groups who share their extreme identity on ritual dimension perceive it as legitimate tactic. The differences along the political dimension can lead them into efforts to dominate each other, and open contests for power.

The consequence of similarity and difference in the dimensions of religious extremism is relevant previous work on identity and conflict (Haslam et al., 1999). In this model, the salience of subgroup identity (e.g., as an activist of PKS or *Hizbut Tahrir*) can lead to a tendency to seek in-group favoritism, which in turn enhances their sense of self. However, when superordinate identity is salient (e.g., as Muslims who support the legalization of Islamic law in Indonesia, or as Muslims in a broader context), in-group members perceive the members of other Islamic movements as members of the same group. According to this, an approach to religious extremism that focuses solely on one dimension will miss the different ways in which the two groups align (e.g., socially) and are different (e.g., politically), which in turn would fail to predict the group members’ political alliances or conflict.

## Applying the Model

To apply this model in more practical uses, we need to revisit the reason of this multidimensional model development. Unidimensional categorization of moderate vs. extremist lead to simplistic understandings whereby people with highly conservative beliefs in religion are associated with support for violence and terror. We propose that extremism is expressed along different dimensions and the mapping of groups and individuals using multiple dimensions in the model will help to understand the patterns of narratives and actions delivered by the groups. This allows for a more nuanced understanding of religious violence whereby we acknowledge that violence can be motivated by different reasons (not necessarily related to political causes) and that the interplay between different dimensions on which extremism can be expressed can either fuel or restrict religious violence (e.g., when a religious group is located at the extremist end of the political dimensions, but collectively shared theological beliefs preclude exercising violence).

Moving away from over-simplified representations of religious groups as politically motivated, the presented framework offers a practical method to understand the multi-faceted nature of extremism. It aims to analyze religion at both a group and individual level, augmenting scholarly understanding of the religious dimensions that may be relevant to enable accurate predictions of violent extremism based on ideological narratives (Kruglanski et al., 2018). Even though the four dimensions of religious extremism that we present here are informed by prior research on extremism and religiosity, the model that we developed is tailored to the context of Indonesian Muslims and their religious movements. When adopting this model in different or wider contexts (e.g., Islamic movements in Pakistan or Egypt, or Christian groups in the Philippines or Northern Ireland), researchers need to think carefully about the transferability of the model.

Practically speaking, when adopting the model in other contexts, researchers need to engage in qualitative exploration of the dimensions religious groups use to express their religiosity. For every dimension found in a particular context, the researchers should then explore what the indicators are of extremism compared to moderate beliefs. Rich descriptive information about the context and specific intra- or intergroup processes need to be considered to enable a multidimensional model tailored and adapted to specific contexts. In this, some dimensions (e.g., ritual, political) may not apply to all contexts, while other new dimensions might need to be added.

Such an exploration may well lead to the conclusion that the political dimension is the most important dimension to explain violent behavior and that the other three proposed dimensions (e.g., theological, social, and ritual) are less relevant. Consider for example the current extremism by Rakhine Buddhist in Myanmar against Rohingya Muslims. Violent actions against Rohingya Muslims in 2017 by Rakhine Buddhist were justified as mere crackdowns against suspected Rohingya insurgents, suggesting that the political dimension may be most important to understand extremism in this context. However, in other contexts, other dimensions appear to have triggered violence. For instance, and also in the context of Buddhist violence, the terrorist sarin attack in the Tokyo subway in 1995 by the cult group Aum Shinrikyo was not so much driven by extremism on the political dimension, but by extremism on the theological and/or ritual dimension. Specifically, the attack was motivated by a strong consensually shared belief among cult members that violence of this form would wash away their sins and this would allow them as a group to survive the imminent Armageddon.

What these examples also make clear is that the content of the different dimensions and the way that moderate vs. extreme religiosity manifests itself differ for different religious groups. Specifically, while it is important to understand political violence among Indonesian Muslims in terms of views on *sharia* laws, in the Myanmar context, political extremism centers on views against minorities and their rights. Or, while theological extremism in Indonesia is concerned with the view of God and ritual extremism relates to tolerance for deviating from generally accepted normative ways of enacting religion, for Aum Shinrikyo in Japan, extremism on these dimensions is related to narratives and beliefs around Doomsday.

Finally, when applying the model in other contexts, it is important to consider new dimensions that may be important in understanding extremism. For example, Smart (1999) identifies seven dimensions of Buddhist religiosity, including novel dimensions such as the mythological and the experiential. Scholars would discover if these dimensions or others are relevant to differences between moderates and extremists (for example, if Buddhist groups who are more mystical are less likely to be extremist) through exploratory research and pilot testing.

We, of course, support the prevention of violent extremism, but we also support the notion that being extreme in religious beliefs is not always linked to support for employing violent tactics (Austin, 2018). Motivating people to participate in violent intergroup conflict, strong narratives about injustice and expected changes may be involved (Moghaddam, 2005; Horgan, 2008). However, in many contexts (i.e., when the conflict involves religious groups), religious narratives can fuel the willingness to join violent movements on behalf of their group. By capturing how extremism is manifested across particular dimensions and how these dimensions predict support for violence, policy-makers can be more focused in countering the religious narratives that might be employed as the catalyst of violence and which are not relevant to address (or even counter-productive).

## Implications

This paper highlights that religious extremism is not a unified and ubiquitous phenomenon; rather, religious extremists differ on a number of dimensions in how they express their religion, and consequently, how they aim to achieve important group goals. Using the context of Indonesian Muslim groups to explore these ideas, we propose that religiosity in Muslims can be moderate on one dimension and radical/extreme on another. For instance, even though the *Salafi* movement has been generally identified as extremist (Haron and Hussin, 2013; Jones, 2014), to understand their extremism, we argue that it is important to be both mindful of the group’s extreme position when considering theological and ritual dimensions, but also their comparatively moderate stand politically. For example, even though Salafi movements in Indonesia perceive politics as morally corrupt (Chozin, 2013; Parveez, 2017), they nevertheless tend to avoid a political debate, and obey the rules of the existing government insofar as the government does not prohibit their religious rituals (Haron and Hussin, 2013; Parveez, 2017).

In a similar vein, the group *Hizb al-tahrir* is extreme in its stance on the political dimension, as it aims to revive the Islamic empire by overthrowing the concept of the nation state (e.g., Ward, 2009; Osman, 2010b). Nevertheless, their activists are moderate on the ritual dimension – they do not criticize other Muslims for their “innovative” rituals (e.g., celebration of the Prophet’s birthday) – and they do not support the use of physical violence in pursuing political demands (See: Ward, 2009; Schmid, 2013; Parveez, 2017). The group believes that *jihad* means a holy war, but not as the way to establish the *Caliphate,* but to conquer other nations after the Caliphate is established (Azman, 2015). In addition, this group was actively involved in protests to reject the cultures and norms of other groups in Indonesia on behalf of Muslim as majority (e.g., rejecting the celebration of Valentine’s day). We might argue that *Hizbut Tahrir* is not only extreme in its political dimension, but also theological and social dimensions. Nevertheless, this group seems to be moderate in the ritual dimension.

Our purpose in this paper is to illustrate that different dimensions of religion are relevant to understanding religious extremism, and that the four dimensions discussed provide clarity in distinguishing a diversity of extreme vs. moderate presentations in the Indonesian Muslim context. Identifying religious extremism as multidimensional helps moving beyond labeling Muslims simply as liberal, extreme, progressive, moderate, or radical. These labels fail to capture the various religious groups’ similarities and differences across different dimensions, and wrongly cluster together religious actors with quite different historical pasts and future trajectories. This “concept creep” (Haslam, 2016) or “jingle-jangle fallacy” (Van Petegem et al., 2013) prevents scholars from identifying the antecedents, character, and consequences of religious extremism in different aspects of life.

We invite scholars to consider extremism in relation to individual and group positions on theological, ritual, social, and political dimensions, and to expect a diversity of contestations within a faith that do not always co-vary. With this approach, it is important to be mindful of the fact that when researchers explore the relationship of religious extremism and other psychological processes, the type (dimension) of extremism needs to be considered. For instance, as seen in the narratives of some extremist groups in Indonesia who highlight the “crisis of Islam” as a call to seek systemic change, we predict that perceived injustice toward the religion by outsiders can enhance extremism on the political dimension, but may not affect extremism on the other dimensions as strongly. In this way, we can advance knowledge of religious extremism, allowing us to move toward a more complete understanding of what is not just one phenomenon, but a constellation of related phenomena in an evolving, complex religious system of beliefs and acts embedded in broader historical and cultural change and stability.

## Conclusion

Labeling groups or individuals as extremist is often misleading. The label has a narrow pejorative meaning which too often associates extremism with terrorism (e.g., the Bali bombings, or the Paris attacks). Failure to understand the complexity of religious extremism risks stigmatizing some religious groups as irrational and supporting of violence when this is not the case. These negative stereotypes can lead to separation, status loss, and discrimination, as well as wasted resources in mis-targeted counter-terrorism initiatives, and squandered political capital. Our hope is that a more comprehensive understanding of religious extremism will facilitate better insight and nuanced dialogue. Understanding the multidimensionality of religion in the context of religious extremism will help in accurately depicting this phenomenon, and will facilitate understanding by scholars of the complex group processes associated with religious change, which have been neglected to date.

## Author Contributions

SW conceived of the presented idea. SW wrote the manuscript with support from WL and JJ. SW, WL, and JJ contributed to the final version of the manuscript, responding to reviewers’ feedback.

### Conflict of Interest

The authors declare that the research was conducted in the absence of any commercial or financial relationships that could be construed as a potential conflict of interest.
